# Changes in Serum Free Amino Acids and Muscle Fatigue Experienced during a Half-Ironman Triathlon

**DOI:** 10.1371/journal.pone.0138376

**Published:** 2015-09-15

**Authors:** Francisco Areces, Cristina González-Millán, Juan José Salinero, Javier Abian-Vicen, Beatriz Lara, Cesar Gallo-Salazar, Diana Ruiz-Vicente, Juan Del Coso

**Affiliations:** Exercise Physiology Laboratory, Sports Science Institute, Camilo José Cela University, Madrid, Spain; University of the Balearic Islands, SPAIN

## Abstract

The aim of this study was to investigate the relationship between changes in serum free amino acids, muscle fatigue and exercise-induced muscle damage during a half-ironman triathlon. Twenty-six experienced triathletes (age = 37.0 ± 6.8 yr; experience = 7.4 ± 3.0 yr) competed in a real half-ironman triathlon in which sector times and total race time were measured by means of chip timing. Before and after the race, a countermovement jump and a maximal isometric force test were performed, and blood samples were withdrawn to measure serum free amino acids concentrations, and serum creatine kinase levels as a blood marker of muscle damage. Total race time was 320 ± 37 min and jump height (-16.3 ± 15.2%, *P* < 0.001) and isometric force (-14.9 ± 9.8%; *P* = 0.007) were significantly reduced after the race in all participants. After the race, the serum concentration of creatine kinase increased by 368 ± 187% (*P* < 0.001). In contrast, the serum concentrations of essential (-27.1 ± 13.0%; *P* < 0.001) and non-essential amino acids (-24.4 ± 13.1%; *P* < 0.001) were significantly reduced after the race. The tryptophan/BCAA ratio increased by 42.7 ± 12.7% after the race. Pre-to-post changes in serum free amino acids did not correlate with muscle performance variables or post-race creatine kinase concentration. In summary, during a half-ironman triathlon, serum amino acids concentrations were reduced by > 20%. However, neither the changes in serum free amino acids nor the tryptophan/BCAA ratio were related muscle fatigue or muscle damage during the race.

## Introduction

Despite its current popularity, the cause(s) of fatigue in the triathlon and its underlying mechanism(s) have only recently begun to be explored. Like in other endurance disciplines, fatigue during triathlon competitions is a complex factor related to both central and peripheral factors that are influenced by the intensity and duration of the race, the nutritional intake during the race, the training status of the triathlete and different environmental conditions [1,2]. In long-distance triathlons (> 60 min of duration), local fatigue has been mainly associated to depletion of muscle glycogen [3] and failure of neuromuscular transmission [4]. Local (e.g., muscle) fatigue in long-distance triathlons has also been related to the ultrastructural damage produced in the muscle fiber by the continuous muscle contractions [5]. In fact, the concentration of blood markers of muscle damage (e.g., myoglobin and creatine kinase) has been related to the decrease in muscle function during a half-ironman triathlon [2,6]. A similar relationship between exercise-induced muscle damage and muscle fatigue has been found in other endurance sport disciplines, such as the marathon [7,8].

The development and extent of fatigue (both local and central) reached during an endurance competition can be affected by the serum concentrations of essential amino acids [9]. Essential amino acids are biomolecules that cannot be synthesized *de novo* by the human body, and therefore they must be supplied in the diet to obtain normal/physiological serum values. Despite the absence of synthesis in the human body, essential amino acids play a crucial role in the protein synthesis and thus, they are necessary for a myriad of physiological functions [10]. Of the essential amino acids, the branched-chain amino acids (BCAA), and leucine in particular, are considered as the most relevant amino acids, especially for exercise physiology. During and after exercise, BCAA intervene in the muscle protein synthesis by stimulating mRNA translation [11] and they prevent muscle proteolysis by inhibiting mechanisms that involve the mammalian target of rapamycin [12,13]. Furthermore, BCAA can be oxidized extrahepatically in the skeletal muscle tissue to produce energy although its contribution to energy expenditure is only noteworthy during endurance exercise [14].

Supplementation of BCAA has proven to be effective to accelerate recovery after exercise-induced muscle damage [15–19] although its effectiveness to improve physical performance is unclear [20–25]. The utilization of serum BCAA can reduce protein turnover in the active muscle and they can be physiologically essential to ameliorate the muscle fatigue experimented during a triathlon once known that muscle damage is directly related to fatigue in this sport [2,6]. To our knowledge, no study has investigated whether the changes in serum BCAA are related to the extent of muscle damage and muscle fatigue attained during prolonged exercise.

Essential amino acids, especially BCAA, can also play a role for reducing central fatigue during endurance exercise [26]. It is well known that exercise raises the release of the neurotransmitter serotonin (5HT) into the brain and for some time it has been hypothesized that 5HT contributes to central fatigue during exercise [27] due to its relationship with sleep and lethargy [9]. The “tryptophan-5HT-central fatigue theory” proposes that an increase in the level of 5-HT in a presynaptic neuron would lead to and increased amount of 5-HT being released into the synapse upon stimulation, ultimately producing central fatigue [26]. According to this theory, the rate of 5HT synthesis is regulated by the concentration of tryptophan in the blood [28]. Thus, an increase in serum tryptophan levels can rise 5HT concentration into the brain, leading to the development of central fatigue. Although this hypothesis has been tested by several investigations [22–25], has also been refuted the interrelationship between increased blood tryptophan and central fatigue [29].

BCAA have been proposed to alleviate central fatigue due to their ability to compete with tryptophan in crossing blood-brain barrier. BCAA use the same transporter as tryptophan to pass through the blood-brain barrier and thus, the tryptophan/BCAA ratio into circulating blood has been suggested as an essential factor for 5HT synthesis [30]. Therefore, the decreased serum tryptophan/BCAA ratio would reduce the cerebral uptake of tryptophan and subsequently, 5HT synthesis [23]. There is evidence that BCAA intake influences physical performance but most investigations that have found a limited 5HT synthesis with BCAA supplementation have been performed on animal models [31,32]. For instance, exercising rats prefer a BCAA-based solution over water during times of intense exercise [31]. In addition, adult male rats fed with a BCAA-fortified diet increased the volume of free physical activity [31]. In horses competing in long distance rides of different lengths, it has been found that the utilization of serum BCAA is more important the longer the distance, while the tryptophan/BCAA ratio was higher in the longer distances races [32]. In humans, the increased availability of serum BCAA induced by pre-exercise BCAA intake did not change sprint [33] or endurance performance [14,22,27] although it increased exercise intensity at lactate threshold [25], upper body muscle power [34] and the rate of perceived recovery after strenuous exercise [24]. In any case, the association between the ergogenic effect of the BCAA administration and the reduction of 5HT production into the brain has not been tested in humans.

The aim of this study was to investigate the relationship between the changes in serum free amino acids and physical performance variables during a half-ironman triathlon. We hypothesized that triathletes with a higher reduction of serum BCAA (measured by pre-to-post-race change in serum BCAA concentration) would have ameliorated muscle damage and muscle fatigue during the race. A second hypothesis was that triathletes with a higher tryptophan/BCAA would have a reduced performance during the race due to the “tryptophan-5HT-central fatigue theory”.

## Methods

### Ethics statement

All the participants were informed of the risks and discomforts associated with the investigation and signed a written consent to participate. The study was specifically approved by the Camilo Jose Cela University Ethics Committee in accordance with the latest version of the Declaration of Helsinki.

### Subjects

Twenty six male healthy and experienced triathletes volunteered to participate in this investigation. Potential participants with a previous history of muscle disorder, cardiac or kidney disease or those taking medicines were discarded. We also discarded participants that had used protein supplements in the two weeks prior to the race. A questionnaire about their medical history, previous training, triathlon experience and best race time in half-ironman triathlon races was filled out by each participant and their main characteristics are shown in Table 1.

**Table 1:** Morphological characteristics, training status and best race time in the half-ironman distance for the participants in this investigation.

*Variable*	*mean ± SD*
n	26
Age (yr)	37.0 ± 6.8
Weight (kg)	74.1 ± 7.5
Height (cm)	177 ± 6
Experience (yr)	7.4 ± 3.0
Swimming training (km/wk)	8.6 ± 5.3
Cycling training (km/wk)	256 ± 131
Running training (km/wk)	50.2 ± 24.8
Best race time (min)	310 ± 32

Swimming, cycling and running training represent the mean distance covered per week during the practices in the month prior to the race.

### Experimental Protocol

Participants were instructed to perform light exercise and to avoid pain-relieving strategies (e.g., analgesic medications, manual massage, ice, etc) the two days before the race. In addition, participants were instructed to avoid any sources of caffeine and alcohol 24 h before the onset of the race. Three hours before the race, participants arrived at an area close to the start line having drunk 500 mL of tap water two hours before arrival to ensure euhydration. Participants had their habitual pre-competition meal before the race at least 2 h before arrival but diet was not standardized to avoid affecting participants’ pre-competition routines. A pre-race food diary was obtained for each participant and was analyzed at a later date (PCN Cesnid, 2.0). All participants presented a proportion of 12–18% of proteins in their 24-h diet before the race.

Participants rested for 10 minutes in a recumbent chair and a 5-mL venous blood sample was drawn from an antecubital vein. The blood was allowed to clot and centrifuged at 5000g to obtain serum. The serum was immediately kept at 4°C until analysis (see below). Then, participants completed a 10-min standardized warm-up and performed two maximal countermovement jumps (CMJ) on a force platform (Quattrojump, Kistler, Switzerland). Participants were previously familiarized with the jump test. After two minutes of rest, participants performed a 5-s whole-body isometric muscle strength test measured by means of a hand-held pull gauge (Isocontrol isometric EV-Pro, Spain). For this measurement, participants stand on a 50 × 50 cm iron base connected to a handle-bar by a non-elastic cable. Participants were instructed to perform a maximal pull using their whole body (mainly legs and arms) and they wore an adjustable lumbar back protector for support and protection during the test. Participants were also previously familiarized with the isometric force test.

Fifteen min before the race, participants were weighed in their competition clothes (without wetsuit; BC-418, Tanita, Japan) and no instruction about drinking or food were given for the race. The race consisted of 1.9 km of swimming, 75 km of cycling and 21.1 km of running and is commonly known as a half-ironman. During the swim section, all participants wore a wetsuit because water temperature was 18 ± 1°C. During the cycling and running sections, ambient temperature was 23 ± 3°C (range = 19–27°C) while relative humidity was 37 ± 8% (range = 32–45%). During the race, participants ingested water, sports drinks and food (mainly fruit and energy bars) *ad libitum* at aid stations but they were instructed to remember the amount and type of food and drink ingested during the race. Participants were instructed to avoid supplements that contained sources of branched-chain amino acids.

Within 1 min of the end of the race, participants went to a finish area and body mass, height during CMJ jumps and a whole-body isometric muscle strength test were measured in this order using the same methodology described previously. Participants then rested for 5 min and a venous blood sample was obtained and allowed to clot. Then, participants filled out a questionnaire that included items about the rate of perceived exertion (Borg scale from 6 to 20 points), perceived leg muscle soreness (visual analog scale from 0 to 10 points) and detailed type and amounts of fluid and food intakes during the race.

The serum portion of each blood sample was analyzed within 48 h for osmolality (1249, Advance 3MO, Spain), creatine kinase concentration (AU5400, Beckman Coulter, US) and serum free amino acid concentration (HPLC; 1290 Infinity II LC Systems, Agilent, Santa Clara, CA), as previously described [35]. The amount of total amino acids, essential amino acids (valine, leucine, isoleucine, threonine, methionine, phenylalanine, lysine, tryptophan and histidine), non-essential amino acids (glycine, alanine, serine, cysteine, asparagine, glutamic acid, glutamine, arginine, tyrosine and proline), branched-chain amino acids (e.g., BCAA = the sum of valine, leucine and isoleucine) and the tryptophan/BCAA ratio were calculated using data from individual serum free amino acid concentrations.

### Statistical Analysis

The normality of each variable was tested with the Shapiro-Wilk test. All the variables presented a normal distribution. Pre-to-post-race changes in all the variables were identified using Student’s t test for paired samples. Pearson’s correlation was used to assess the association between two variables. The data were analyzed with the SPSS statistical package version 18.0 (SPSS Inc., Chicago, IL) and the significance level was set at *P* < 0.05. Data are presented as mean ± SD throughout the text.

## Results

The mean race time during the half-ironman triathlon was 320 ± 37 min. By sectors, participants took 42.1 ± 8.3 min to complete the swimming, 158 ± 16 min to complete the cycling and 112 ± 18 min to complete the running sections. These times represent 13.2, 49.3 and 34.9% of the total time employed to finish the race. From 30.5 ± 5.1 cm, maximal height during CMJ was reduced to 25.2 ± 4.9 cm after the race, representing a mean reduction of 16.3 ± 15.2% (*P* < 0.001). Similarly, maximal isometric strength was reduced from 1297 ± 301 to 1104 ± 257 N after the race which represents a mean reduction of 14.9 ± 9.8% (*P* = 0.007). During the race, body mass changed from 74.1 ± 7.5 to 71.8 ± 7.1 kg which represents a pre-to-post-race change of -3.1 ± 1.2% (*P* < 0.001). After the race, the rate of perceived exertion was 16 ± 2 points and perceived leg muscle soreness was 6 ± 1 points. During the race, participants ingested 1730 ± 546 mL of fluid and 832 ± 355 kcal. From the total energy intake, 192 ± 82 g were carbohydrates, 1.8 ± 0.9 g were fats and 0.1 ± 0.1 g were proteins.

Serum osmolality and serum concentrations of total amino acids, essential and non-essential amino acids before and just after the race are presented in Table 2. Blood serum osmolality increased by 3.8 ± 2.0% during the race (*P* < 0.001). However, the serum concentrations of total amino acids, essential amino acids and non-essential amino acids were significantly reduced by < 20% during the race (*P* < 0.001, Table 2). Similarly, the serum concentration of BCAA was reduced by -32.8 ± 15.6% after the race (*P* < 0.001). Nevertheless, the tryptophan/BCAA ratio significantly increased from pre-to-post-race (*P* = 0.001). The creatine kinase concentration greatly increased during the half-ironman since post-race values were almost four-fold the ones presented before the race (173 ± 72 *vs* 808 ± 502 U/L; *P* < 0.001).

**Table 2:** Changes in serum variables before (pre) and after (post) a half-ironman race.

*Variable (units)*	Pre	Post	Δ	P value
Serum osmolality (mOsm/kg)	291 ± 4.9	302 ± 5	+3.8 ± 2.0	< 0.001
Total amino acids (nmol/mL)	3809 ± 483	2808 ± 396	-25.4 ± 12.6	< 0.001
Essential amino acids (nmol/mL)	1250 ± 173	898 ± 126	-27.1 ± 13.0	< 0.001
Non-essential amino acids (nmol/mL)	2559 ± 350	1910 ± 281	-24.4 ± 13.1	< 0.001
Branched-chain amino acids (nmol/mL)	519 ± 95	340 ± 63	-32.8 ± 15.6	< 0.001
Tryptophan/BCAA ratio (%)	8.6 ± 2.0	11.5 ± 3.2	+42.7 ± 12.7	= 0.001
Tyrosine/BCAA ratio (%)	15.5 ± 4.2	23.3 ± 3.8	+62.4 ± 13.6	< 0.001
Creatine kinase (U/L)	173 ± 72	808 ± 502	+368 ± 187	< 0.001

**Fig 1:**
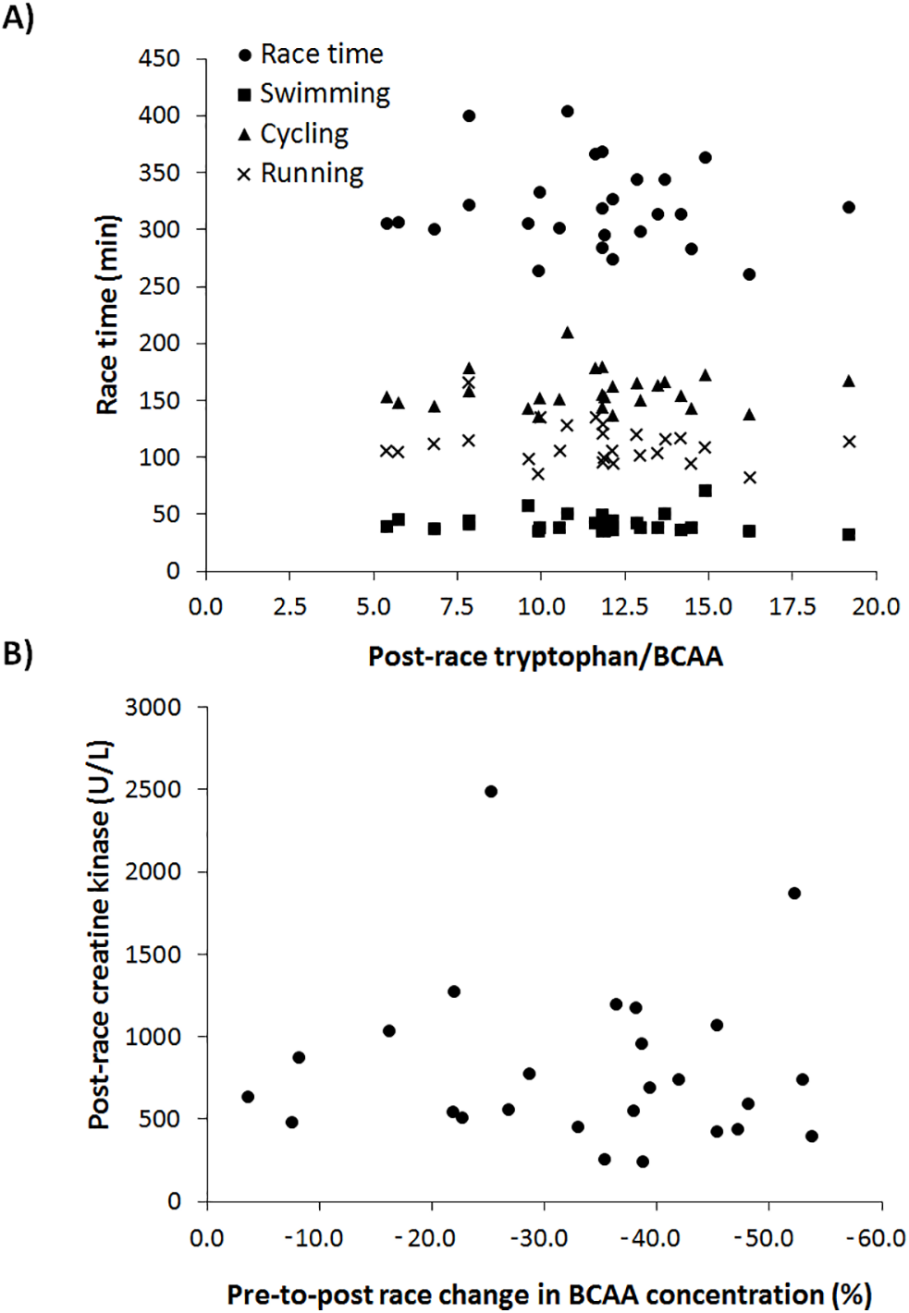
A) Relationship between race time and sector times (swimming, cycling and running) and post-race tryptophan/BCAA ratio during a half-ironman race. B) Relationship between post-race creatine kinase concentration and changes of BCAA concentration during a half-ironman race (as a change from pre-exercise values). Note: A) The correlation of total race time (r = -0.05; *P* = 0.80), swimming time (r = -0.05; *P* = 0.80), cycling time (r = 0.08; *P* = 0.70) and running time (r = -0.21; *P* = 0.30) with post-race tryptophan/BCAA ratio were not statistically significant. B) The correlation of post-race creatine kinase concentration with BCAA change during the race (r = -0.02; *P* = 0.92) was not statistically significant.

Table 3 presents the serum concentration of individual amino acids before and after the half-ironman race. Except for phenylalanine and methionine, the serum free concentrations of all essential amino acids were significantly reduced after the race (*P* < 0.001). Serum free tryptophan concentration tended to be reduced after the race although this change was not statistically significant (*P* = 0.070) and was minor when compared to other essential amino acids. The individual serum free concentration of non-essential amino acids was reduced after the race although tyrosine concentration remained unchanged and cysteine concentration increased by 64.1 ± 84.9% (*P* < 0.001). Race time negatively correlated to post-race blood serum osmolality (r = -0.57; *P* = 0.002) and to CMJ change (r = -0.55; *P* = 0.003). However, race time did not correlate to any change in serum amino acid concentration or the post-race tryptophan/BCAA ratio (Fig 1A). In addition, the changes in serum amino acid concentration did not correlate to any muscle performance variable. Finally, pre-to-post-race changes of BCAA during the race did not correlate to post-race creatine kinase concentration (Fig 1B).

**Table 3:** Serum free amino acid concentration before (pre) and after (post) a half-ironman race.

*Amino acid (units)*	Pre	Post	Δ	P value
*Essential amino acids*				
Valine (nmol/mL)	267 ± 48	170 ± 23	-34.8 ± 11.2	< 0.001
Leucine (nmol/mL)	174 ± 42	112 ± 34	-32.3 ± 27.0	< 0.001
Isoleucine (nmol/mL)	78 ± 18	58 ± 16	-22.6 ± 24.0	< 0.001
Threonine (nmol/mL)	146 ± 27	85 ± 23	-41.0 ± 16.4	< 0.001
Methionine (nmol/mL)	28 ± 6	27 ± 6	-6.1 ± 9.4	= 0.416
Phenylalanine (nmol/mL)	105 ± 20	109 ± 17	+7.0 ± 5.8	= 0.458
Lysine (nmol/mL)	136 ± 37	76 ± 21	-40.9 ± 19.9	< 0.001
Tryptophan (nmol/mL)	44 ± 9	39 ± 11	-7.9± 11.9	= 0.070
Histidine (nmol/mL)	272 ± 33	223 ± 33	-16.9 ± 16.6	< 0.001
*Non-essential amino acids*				
Glycine (nmol/mL)	334 ± 97	220 ± 53	-29.7 ± 27.6	< 0.001
Alanine (nmol/mL)	538 ± 98	312 ± 94	-41.6 ± 16.1	< 0.001
Serine (nmol/mL)	151 ±31	105 ± 22	-28.8 ± 16.5	< 0.001
Cysteine (nmol/mL)	62 ± 23	90 ± 33	+64.1 ± 84.9	= 0.001
Asparagine (nmol/mL)	47 ± 11	31 ± 8	-32.0 ± 20.7	< 0.001
Glutamic acid (nmol/mL)	71 ± 22	58 ± 17	-14.0 ± 28.0	= 0.002
Glutamine (nmol/mL)	1088 ± 134	891 ± 131	-16.9 ± 16.6	< 0.001
Arginine (nmol/mL)	117 ± 17	73 ± 18	-36.5 ± 17.2	< 0.001
Tyrosine (nmol/mL)	77 ± 13	79 ± 18	+6.1 ± 5.2	= 0.696
Proline (nmol/mL)	26 ± 11	18 ± 7	-20.5 ± 40.1	= 0.003

## Discussion

The aim of this research was to investigate the role of serum free amino acids (mainly BCAA and tryptophan) in the development of muscle fatigue and muscle damage during a half-ironman distance triathlon. For this purpose, we measured serum concentrations of free amino acids before and just after the race and we related the changes in these amino acids with specific tests to assess muscle fatigue (jump height and maximal isometric strength) and to blood markers of muscle damage (creatine kinase). The main outcomes were: a) the concentrations of essential (-27.1 ± 13.0%) and non-essential amino acids (-24.4 ± 13.1%) were significantly reduced after the race with BCAA being the group of amino acids that presented a higher reduction (Table 2); b) the tryptophan/BCAA ratio increased by 42.7 ± 12.7% after the race because the serum BCAA concentration was more reduced than the serum concentration of free tryptophan (Table 3); c) after the race, jump height (-16.3 ± 15.2%) and maximal isometric strength (-14.2 ± 9.8%) were significantly reduced while serum creatine kinase concentration increased ≈4 fold. However, these muscle performance decrements were unrelated to the changes of serum free amino acids produced during the race or to the increase in the tryptophan/BCAA ratio (Fig 1A). These data suggest that serum free amino acid are used by active and non-active muscles during prolonged exercise (> 300 min) but their changes did not play a major role in prevention of muscle fatigue or muscle damage during a long-distance triathlon. Besides, a lower tryptophan/BCAA, an indirect indicator of reduced central fatigue, did not prevent muscle fatigue during the race, as measured by pre-to-post race changes in jump height and isometric force.

Based on the high concentric and eccentric muscle demands of the three disciplines in the triathlon (swimming, cycling and running) and the duration of the race, several investigations have reported that ironman and half-ironman triathlon races lead to severe skeletal muscle damage [6,36,37]. Although most investigations indicate that exercise-induced muscle damage is mechanic in nature and mainly produced during the running leg [6], other investigations indicate that lipid peroxidation can also play a role in the development of muscle damage in endurance events [38]. The main performance outcome derived from muscle fiber damage is the reduction of active muscle function as found during jump tests [6,37] and muscular strength tests [4,37,39]. The breakdown of body protein during endurance exercise has been found to produce the mobilization and oxidation of amino acids in muscle fiber [40] as has been indirectly confirmed by the reduction in skeletal muscle mass found in ultra-endurance triathletes [41]. Supplementation with BCAA to increase the serum concentration of this type of amino acids has been suggested as a strategy to prevent muscle damage because of its effectiveness to promote protein synthesis [11] and to preclude proteolysis [12]. Although BCAA supplementation has been effective to reduce the symptoms of exercise-induced muscle damage in the days after the exercise protocol [15–19], its effectiveness during competition has been recently questioned [22].

In the present investigation, the concentrations in all amino acid categories were significantly reduced from pre-to-post race, although BCAA was the group of amino acids with the highest reduction during the race (Table 2). The reduction in the concentration of amino acids during the race was probably produced by the uptake and oxidation of free amino acids (mainly into the active skeletal muscle) since blood was concentrated (as indicated by the increase in blood serum osmolality) and participants minimally ingested proteins during the race. These reductions suggest that serum free amino acids were used as a source of energy for muscle contraction while BCAA were likely the most used amino acid for oxidation during exercise [14]. However, the changes (e.g., reductions) of serum BCAA concentration during the whole race was not related to race time, sector times or the maintenance of jump height and/or maximal isometric strength. In addition, the changes in serum BCAA was not associated with a reduction in the concentration of blood creatine kinase (Fig 1B), an indirect marker of muscle damage. All this information proposes that, despite BCAA constituting an energy source during a half-ironman race, the oxidation of these endogenous amino acids was not related to a better physical performance or to reduced muscle damage during the triathlon.

During endurance exercise, free fatty acids (FFA) are mobilized from adipose tissue and are transported via the blood to the muscle to serve as a fuel for muscle contraction. Both FFA and tryptophan compete for binding to albumin and thus, the increase in FFA serum concentration produced during prolonged exercise prevents/limits the binding of tryptophan and albumin, increasing the concentration of serum free tryptophan. On the other hand, the uptake of BCAA by the active muscle as a source of energy produces that the tryptophan/BCAA ratio greatly increases during exercise [42]. According to the “tryptophan-5HT central fatigue hypothesis” the increase of the tryptophan/BCAA ratio in the blood results in a higher transport of tryptophan across the blood-brain barrier because BCAA and tryptophan compete for the same large neutral amino acid (LNAA) transporter [43]. Thus, an enhanced entry of tryptophan into the brain would lead to increased 5HT levels in specific areas of the brain facilitating the release of 5HT from some neurons, ultimately leading to anticipated central fatigue [42]. Under this paradigm, the intake of BCAA to increase the serum concentration of BCAA would tryptophan/BCAA ratio to decrease the transport of free tryptophan into the brain, reducing the 5HT releasing into synapsis and alleviating central fatigue in several sport disciplines [14,25,44].

The majority of classic and more recent studies, using various exercise protocols and several forms of BCAA administration, have not found any performance benefits derived from BCAA supplementation [21,33,45,46], although this is not always the case [23–25]. The present investigation confirms that the tryptophan/BCAA ratio is increased during a half-ironman triathlon, although most of this effect was due to a reduced serum BCAA concentration rather than increased serum free tryptophan (see Table 3). However, the post-race tryptophan/BCAA ratio and the pre-to-post changes in this ratio were not associated to race time (Fig 1A) or jump height and maximal isometric strength reductions. Although our investigation did not include BCAA supplementation, it suggests that participants that maintained a low blood tryptophan/BCAA ratio did not maintain better muscle function nor did they present an improved overall performance. In fact, the absence of relationship between the tryptophan/BCAA ratio and performance variables can be related to the low level of central fatigue found after the completion of a half-ironman race [6].

The current investigation presents some limitations with respect to the experimental design and the measurements obtained. First, this investigation compares the physiological responses of 26 experienced triathletes with different ages, previous training, and nutritional strategies during the race and thus, it includes some biological variance that could have affected the outcomes of the research. Second, the investigation was performed in an ecologically-valid context and participants were not supplemented with sources of BCAA before or during the race. It is possible that BCAA effectiveness on increasing performance and/or preventing muscle damage during exercise is present with higher serum BCAA concentrations than the ones found without supplementation. Third, there is a lack of physiological measurements during the recovery process of the marathon race. The origin of the participants hindered the obtaining of blood samples and muscle performance measurements in the subsequent days after the race. Despite of these limitations, this investigation suggests that, at “normal” serum concentrations, the role of serum free amino acids and serum BCAA is not crucial for endurance (triathlon) performance.

## Conclusions

In summary, the serum concentrations of essential and non-essential amino acids were reduced during a half-ironman triathlon suggesting that this sport discipline relies in part on amino acids as a substrate for energy production. Furthermore, BCAA were the type of amino acids that presented the highest reduction during the race. The tryptophan/BCAA ratio was significantly increased during the race which might indicate that serotonin synthesis was facilitated during the last stages of the race. However, participants that maintained serum BCAA concentrations or presented a reduced tryptophan/BCAA ratio during the race did not prevent muscle fatigue or muscle damage during the race.

## References

[1] Millet GP, Bentley DJ, Vleck VE (2007) The relationships between science and sport: application in triathlon. Int J Sports Physiol Perform 2: 315–322. 19168931 10.1123/ijspp.2.3.315

[2] Del Coso J, Gonzalez C, Abian-Vicen J, Salinero Martin JJ, Soriano L, Areces F, et al (2014) Relationship between physiological parameters and performance during a half-ironman triathlon in the heat. J Sports Sci 32: 1680–1687. 10.1080/02640414.2014.915425 24825571

[3] Jeukendrup AE (2011) Nutrition for endurance sports: marathon, triathlon, and road cycling. J Sports Sci 29 Suppl 1: S91–99. 10.1080/02640414.2011.610348 21916794

[4] Garcia-Manso JM, Rodriguez-Ruiz D, Rodriguez-Matoso D, de Saa Y, Sarmiento S, Quiroga M (2011) Assessment of muscle fatigue after an ultra-endurance triathlon using tensiomyography (TMG). J Sports Sci 29: 619–625. 10.1080/02640414.2010.548822 21391085

[5] Del Coso J, Areces F, Salinero JJ, Gonzalez-Millan C, Abian-Vicen J, Soriano L, et al (2014) Compression stockings do not improve muscular performance during a half-ironman triathlon race. Eur J Appl Physiol 114: 587–595. 10.1007/s00421-013-2789-2 24337671

[6] Del Coso J, Gonzalez-Millan C, Salinero JJ, Abian-Vicen J, Soriano L, Garde S, et al (2012) Muscle damage and its relationship with muscle fatigue during a half-iron triathlon. PLoS One 7: e43280 10.1371/journal.pone.0043280 22900101 PMC3416828

[7] Del Coso J, Fernandez de Velasco D, Abian-Vicen J, Salinero JJ, Gonzalez-Millan C, Areces F, et al (2013) Running pace decrease during a marathon is positively related to blood markers of muscle damage. PLoS One 8: e57602 10.1371/journal.pone.0057602 23460881 PMC3583862

[8] Del Coso J, Salinero JJ, Abian-Vicen J, Gonzalez-Millan C, Garde S, Vega P, et al (2013) Influence of body mass loss and myoglobinuria on the development of muscle fatigue after a marathon in a warm environment. Appl Physiol Nutr Metab 38: 286–291. 10.1139/apnm-2012-0241 23537020

[9] Roelands B, de Koning J, Foster C, Hettinga F, Meeusen R (2013) Neurophysiological determinants of theoretical concepts and mechanisms involved in pacing. Sports Med 43: 301–311. 10.1007/s40279-013-0030-4 23456493

[10] Maughan RJ, Shirreffs SM (2012) Nutrition for sports performance: issues and opportunities. Proc Nutr Soc 71: 112–119. 10.1017/S0029665111003211 22000743

[11] Anthony JC, Yoshizawa F, Anthony TG, Vary TC, Jefferson LS, Kimball SR (2000) Leucine stimulates translation initiation in skeletal muscle of postabsorptive rats via a rapamycin-sensitive pathway. J Nutr 130: 2413–2419. 11015466 10.1093/jn/130.10.2413

[12] Tang FC (2006) Influence of branched-chain amino acid supplementation on urinary protein metabolite concentrations after swimming. J Am Coll Nutr 25: 188–194. 16766776 10.1080/07315724.2006.10719531

[13] Lynch CJ, Halle B, Fujii H, Vary TC, Wallin R, Damuni Z, et al (2003) Potential role of leucine metabolism in the leucine-signaling pathway involving mTOR. Am J Physiol Endocrinol Metab 285: E854–863. 12812918 10.1152/ajpendo.00153.2003

[14] Greer BK, White JP, Arguello EM, Haymes EM (2011) Branched-chain amino acid supplementation lowers perceived exertion but does not affect performance in untrained males. J Strength Cond Res 25: 539–544. 10.1519/JSC.0b013e3181bf443a 20386134

[15] Nosaka K, Sacco P, Mawatari K (2006) Effects of amino acid supplementation on muscle soreness and damage. Int J Sport Nutr Exerc Metab 16: 620–635. 17342883 10.1123/ijsnem.16.6.620

[16] Shimomura Y, Yamamoto Y, Bajotto G, Sato J, Murakami T, Shimomura N, et al (2006) Nutraceutical effects of branched-chain amino acids on skeletal muscle. J Nutr 136: 529S–532S. 16424141 10.1093/jn/136.2.529S

[17] Howatson G, Hoad M, Goodall S, Tallent J, Bell PG, French DN (2012) Exercise-induced muscle damage is reduced in resistance-trained males by branched chain amino acids: a randomized, double-blind, placebo controlled study. J Int Soc Sports Nutr 9: 20 10.1186/1550-2783-9-20 22569039 PMC3395580

[18] Greer BK, Woodard JL, White JP, Arguello EM, Haymes EM (2007) Branched-chain amino acid supplementation and indicators of muscle damage after endurance exercise. Int J Sport Nutr Exerc Metab 17: 595–607. 18156664 10.1123/ijsnem.17.6.595

[19] Coombes JS, McNaughton LR (2000) Effects of branched-chain amino acid supplementation on serum creatine kinase and lactate dehydrogenase after prolonged exercise. J Sports Med Phys Fitness 40: 240–246. 11125767

[20] Koba T, Hamada K, Sakurai M, Matsumoto K, Hayase H, Imaizumi K, et al (2007) Branched-chain amino acids supplementation attenuates the accumulation of blood lactate dehydrogenase during distance running. J Sports Med Phys Fitness 47: 316–322. 17641599

[21] Knechtle B, Knechtle P, Mrazek C, Senn O, Rosemann T, Imoberdorf R, et al (2011) No effect of short-term amino acid supplementation on variables related to skeletal muscle damage in 100 km ultra-runners—a randomized controlled trial. J Int Soc Sports Nutr 8: 6 10.1186/1550-2783-8-6 21473783 PMC3079604

[22] Areces F, Salinero JJ, Abian-Vicen J, González-Millán C, Gallo-Salazar C, Ruiz-Vicente D, et al (2014) A 7-day oral supplementation with branched-chain amino acids was ineffective to prevent muscle damage during a marathon. Amino Acids 46: 1169–1176. 10.1007/s00726-014-1677-3 24477835

[23] Chang CK, Chang Chien KM, Chang JH, Huang MH, Liang YC, Liu TH (2015) Branched-chain amino acids and arginine improve performance in two consecutive days of simulated handball games in male and female athletes: a randomized trial. PLoS One 10: e0121866 10.1371/journal.pone.0121866 25803783 PMC4372381

[24] Hsu MC, Chien KY, Hsu CC, Chung CJ, Chan KH, Su B (2011) Effects of BCAA, arginine and carbohydrate combined drink on post-exercise biochemical response and psychological condition. Chin J Physiol 54: 71–78. 21789887 10.4077/cjp.2011.amk075

[25] Matsumoto K, Koba T, Hamada K, Tsujimoto H, Mitsuzono R (2009) Branched-chain amino acid supplementation increases the lactate threshold during an incremental exercise test in trained individuals. J Nutr Sci Vitaminol (Tokyo) 55: 52–58. 19352063 10.3177/jnsv.55.52

[26] Newsholme EA, Blomstrand E (2006) Branched-chain amino acids and central fatigue. J Nutr 136: 274S–276S. 16365097 10.1093/jn/136.1.274S

[27] Cheuvront SN, Carter R 3rd, Kolka MA, Lieberman HR, Kellogg MD, Sawka MN (2004) Branched-chain amino acid supplementation and human performance when hypohydrated in the heat. J Appl Physiol (1985) 97: 1275–1282. 15358751 10.1152/japplphysiol.00357.2004

[28] Meeusen R, Watson P (2007) Amino acids and the brain: do they play a role in "central fatigue"? Int J Sport Nutr Exerc Metab 17 Suppl: S37–46. 18577773 10.1123/ijsnem.17.s1.s37

[29] Fernstrom JD, Fernstrom MH (2006) Exercise, serum free tryptophan, and central fatigue. J Nutr 136: 553S–559S. 16424146 10.1093/jn/136.2.553S

[30] Blomstrand E, Celsing F, Newsholme EA (1988) Changes in plasma concentrations of aromatic and branched-chain amino acids during sustained exercise in man and their possible role in fatigue. Acta Physiol Scand 133: 115–121. 3227900 10.1111/j.1748-1716.1988.tb08388.x

[31] Smriga M, Kameishi M, Torii K (2006) Exercise-dependent preference for a mixture of branched-chain amino acids and homeostatic control of brain serotonin in exercising rats. J Nutr 136: 548S–552S. 16424145 10.1093/jn/136.2.548S

[32] Assenza A, Bergero D, Tarantola M, Piccione G, Caola G (2004) Blood serum branched chain amino acids and tryptophan modifications in horses competing in long-distance rides of different length. J Anim Physiol Anim Nutr (Berl) 88: 172–177. 15059243 10.1111/j.1439-0396.2004.00493.x

[33] Nelson AR, Phillips SM, Stellingwerff T, Rezzi S, Bruce SJ, Breton I, et al (2012) A protein-leucine supplement increases branched-chain amino acid and nitrogen turnover but not performance. Med Sci Sports Exerc 44: 57–68. 10.1249/MSS.0b013e3182290371 21685813

[34] Crowe MJ, Weatherson JN, Bowden BF (2006) Effects of dietary leucine supplementation on exercise performance. Eur J Appl Physiol 97: 664–672. 16265600 10.1007/s00421-005-0036-1

[35] Cooper C, Packer N, Williams K (2001) Amino acid analysis protocols Totowa, NJ: Springer Science & Business Media.

[36] Burger-Mendonca M, Bielavsky M, Barbosa FC (2008) Liver overload in Brazilian triathletes after half-ironman competition is related muscle fatigue. Ann Hepatol 7: 245–248. 18753992

[37] Suzuki K, Peake J, Nosaka K, Okutsu M, Abbiss CR, Surriano R, et al (2006) Changes in markers of muscle damage, inflammation and HSP70 after an Ironman Triathlon race. Eur J Appl Physiol 98: 525–534. 17031693 10.1007/s00421-006-0296-4

[38] Stepanyan V, Crowe M, Haleagrahara N, Bowden B (2014) Effects of vitamin E supplementation on exercise-induced oxidative stress: a meta-analysis. Appl Physiol Nutr Metab 39: 1029–1037. 10.1139/apnm-2013-0566 25068790

[39] Margaritis I, Tessier F, Verdera F, Bermon S, Marconnet P (1999) Muscle enzyme release does not predict muscle function impairment after triathlon. J Sports Med Phys Fitness 39: 133–139. 10399422

[40] Dohm GL, Tapscott EB, Kasperek GJ (1987) Protein degradation during endurance exercise and recovery. Med Sci Sports Exerc 19: S166–171. 3316916

[41] Mueller SM, Anliker E, Knechtle P, Knechtle B, Toigo M (2013) Changes in body composition in triathletes during an Ironman race. Eur J Appl Physiol 113: 2343–2352. 10.1007/s00421-013-2670-3 23748466

[42] Blomstrand E (2006) A role for branched-chain amino acids in reducing central fatigue. J Nutr 136: 544S–547S. 16424144 10.1093/jn/136.2.544S

[43] Chaouloff F, Laude D, Elghozi JL (1989) Physical exercise: evidence for differential consequences of tryptophan on 5-HT synthesis and metabolism in central serotonergic cell bodies and terminals. J Neural Transm 78: 121–130. 2478662 10.1007/BF01252498

[44] Gleeson M (2005) Interrelationship between physical activity and branched-chain amino acids. J Nutr 135: 1591S–1595S. 15930475 10.1093/jn/135.6.1591S

[45] Varnier M, Sarto P, Martines D, Lora L, Carmignoto F, Leese GP, et al (1994) Effect of infusing branched-chain amino acid during incremental exercise with reduced muscle glycogen content. Eur J Appl Physiol Occup Physiol 69: 26–31. 7957152 10.1007/BF00867923

[46] van Hall G, Raaymakers JS, Saris WH, Wagenmakers AJ (1995) Ingestion of branched-chain amino acids and tryptophan during sustained exercise in man: failure to affect performance. J Physiol 486 (Pt 3): 789–794. 7473239 10.1113/jphysiol.1995.sp020854 PMC1156566

